# Comparative Safety of Ustekinumab and Vedolizumab in Older Patients with Inflammatory Bowel Disease: A Bicentric Cohort Study

**DOI:** 10.3390/jcm11236967

**Published:** 2022-11-25

**Authors:** Alice Burgevin, Bénédicte Caron, Alexa Sasson, Amandine Luc, Patrick Netter, Cédric Baumann, Ashwin N. Ananthakrishnan, Laurent Peyrin-Biroulet

**Affiliations:** 1 Department of Gastroenterology, University Hospital of Nancy, F-54000 Nancy, France; 2 NGERE (Nutrition-Génétique et Exposition aux Risques Environnementaux), U1256 INSERM, University of Lorraine, F-54000 Nancy, France; 3 Division of Gastroenterology, Massachusetts General Hospital and Harvard Medical School, Boston, MA 02114, USA; 4 Unit of Methodology, Data Management and Statistic, University Hospital of Nancy, F-54000 Vandœuvre-lès-Nancy, France; 5 Ingénierie Moléculaire et Ingénierie Articulaire (IMoPA), UMR-7365 CNRS, Faculté de Médecine, University Hospital of Nancy, University of Lorraine, F-54000 Vandoeuvre-lès-Nancy, France

**Keywords:** Crohn’s disease, ulcerative colitis, older, biologics, safety

## Abstract

Introduction: Data about the safety of vedolizumab and ustekinumab are lacking in older patients with inflammatory bowel disease. The objective was to compare the safety of vedolizumab and ustekinumab therapies in older patients (>60 years) with inflammatory bowel disease. Methods: This retrospective study included patients with Crohn’s disease or ulcerative colitis initiating vedolizumab, ustekinumab or anti-TNF therapy at >60 years of age. We examined the occurrence of adverse events within one year after therapy. Results: This study included 182 patients: 53 were treated with vedolizumab (22 patients with Crohn’s disease and 31 with ulcerative colitis), 31 with ustekinumab (30 Crohn’s disease and one ulcerative colitis) and 98 with anti-TNF (63 Crohn’s disease and 35 ulcerative colitis). At one year, there was no difference in terms of safety in patients with Crohn’s disease between vedolizumab and ustekinumab considering the number of adverse events per year of follow-up (*p* = 0.258). For ulcerative colitis and Crohn’s disease, the occurrence of adverse events per year of follow-up was similar between vedolizumab and anti-TNF (*p* = 0.274 and *p* = 0.876, respectively). Conclusions: Safety was similar between vedolizumab and ustekinumab in older patients with inflammatory bowel disease.

## 1. Introduction

Inflammatory bowel diseases (IBDs), mainly comprising Crohn’s disease (CD) and ulcerative colitis (UC), are chronic immune-mediated diseases predominantly affecting the gastrointestinal tract that generally begin in young adulthood and last throughout life [1]. Between 10 and 30% of patients with IBD are diagnosed at an age of over 60 years [2,3].

Therapeutic options include conventional treatments (corticosteroids, 5-aminosalicylates and immunosuppressants) and biologics [4]. Vedolizumab (VDZ) is a monoclonal antibody that targets the α4β7 integrin and selectively prevents the infiltration of leucocytes into the gastrointestinal submucosa. Ustekinumab (UST) is a human IgG antibody targeting the p40 subunit of IL-12 and IL-23,17 [5,6]. These drugs were introduced for the treatment of IBD, offering an alternative treatment option with a different mechanism of action than, for example, anti-tumor necrosis factor (TNF) therapy. Both VDZ and UST have displayed favorable safety profiles in randomized controlled trials and observational cohorts [7,8,9,10].

Due to the differences in disease phenotype and their potential comorbidity, the management of older patients with IBD (>60 years) might be different [11]. Only few studies have focused on the management of older patients with these drugs [12,13], as older patients or patients with significant comorbidities are often excluded from clinical trials [3]. In a systematic review and meta-analysis, an almost threefold higher risk of serious infections was observed among older patients with IBD exposed to biologics compared to the unexposed [4]. For opportunistic infections, the authors identified a three times higher risk among older patients exposed to biologics. In this study, there was no evidence of an increased risk of malignancies [4]. The majority of evidence about the safety of biologics in older patients with IBD concerns anti-TNF. There are very limited data for anti-integrins [14,15,16], and UST [17].

The objective of this study was to assess the safety of VDZ and UST therapy in older patients with IBD.

## 2. Materials and Methods

### 2.1. Study Population

We conducted a retrospective observational multicenter study in two centers, the Nancy University Hospital (France) and the Massachusetts General Hospital (Boston, MA, USA), including consecutive older patients with IBD who started treatment with UST, VDZ or anti-TNF from June 2014 to May 2020.

Patients were eligible if they were more than 60 years old at the time of the first injection of treatment, had a confirmed diagnosis of UC or CD, had received at least one injection of VDZ, UST or anti-TNF and were followed for at least one year. Inclusion date corresponded to the first infusion of VDZ or UST. Drugs were used according to licensed or published doses and frequency. UST was started by a first 6 mg/kg infusion at inclusion, followed by maintenance regimen. VDZ was started by three 300 mg infusions at weeks 0, 2 and 6 followed by infusions every 8 weeks as maintenance treatment.

### 2.2. Data Collection

Patients’ medical records were retrospectively reviewed and the following data at inclusion were collected through a standardized form: demographic characteristics, comorbidities, smoking status, disease duration and IBD phenotype according to the Montreal classification and medical and surgical treatment history. Adverse events were collected.

### 2.3. Outcomes Measures

The primary endpoint was the safety of VDZ and UST assessed by the occurrence of adverse events (AEs) (infections, cancers or any other adverse event, such as dermatological pathology, allergic reaction and rheumatic manifestation) leading or not leading to drug withdrawal. The follow-up period started at inclusion until treatment discontinuation or end of the follow-up period in December 2020. Comparative safety between VDZ or UST and TNF-treated patients was assessed as secondary endpoints.

### 2.4. Statistical Analysis

Safety data were reported based on patient years of exposure. First, categorical variables were described by %, and continuous variables by median +/− standard deviation. Comparisons by treatment were realized using the Chi-2 or Fisher test for qualitative variables and the Wilcoxon test for quantitative variables. Propensity score (PS) was calculated as the predicted probability of starting treatment with VDZ versus UST, conditioned on the measured baseline variables thought to be confounders or predictors for the outcome of interest. The variables that we used for the main PS model were age at diagnosis, sex, disease duration, ano-perineal disease, prior TNF-antagonist exposure and prior exposition to VDZ or UST. Data used in the patient with UC propensity score included gender, smoking status, exposure at baseline to corticosteroids, azathioprine and methotrexate. Finally, safety of treatments was compared using an inverse PS adjusted generalized linear model. All statistical analyses were performed using SAS software (version 9.4, SAS Institute Inc., Cary, NC, USA).

## 3. Results

### 3.1. Baseline Characteristics

A total of 182 patients were included: 53 were treated with VDZ, 31 with UST and 98 with anti-TNF. Baseline characteristics are reported in Table 1 and Table 2. The mean age at first injection was similar in the three groups for patients with CD or UC. The median duration of the disease at inclusion was similar in all three groups for both patients with CD and patients with UC. There was a female predominance for patients with CD in the UST group (70%) versus the VDZ group (50%) and the anti-TNF group (44%). Steroid dependence at baseline was higher in the anti-TNF group for patients with CD. There was no significant difference for concomitant immunosuppressants during treatment with biologics in all three groups for patients with CD and UC. No significant difference in the distribution of comorbidities was observed between the three groups for patients with CD, and between the two groups for patients with UC.

### 3.2. Safety Outcome

The safety of VDZ compared to UST could only be assessed in patients with CD, given the absence of patients with UC in the UST group. A total of four AEs per year of follow-up occurred in the VDZ group and seven AEs per year of follow-up occurred in the UST group. No severe AEs or discontinuation of treatment were reported. There was no significant difference in terms of the safety between the two groups (*p* = 0.258) (Table 3). The most common AEs were infectious complications (pyelonephritis, urinary tract infections, pneumonia, shingles, infectious diarrhea or perianal abscesses), allergic reaction to infusion and dermatological manifestations (hair loss or non-specific skin rash). No neoplastic complication was reported in both groups (Table 4).

When comparing older patients with CD treated with UST or anti-TNF, 7 AEs per year of follow-up occurred in UST-treated patients and 12 in anti-TNF-treated patients. No severe AEs or discontinuation of treatment were reported. There was no significant difference in terms of the safety between these two groups (*p* = 0.32). (Table 3). The described AEs were infectious (pyelonephritis, urinary tract infections, pneumonia or infectious diarrhea), rheumatological (joint pains) and dermatological (hair loss, non-specific skin rash or paradoxical psoriasis). Patients treated with UST mainly developed infectious complications, whereas patients treated with anti-TNF mostly developed rheumatic and dermatological complications (Table 5).

For patients with CD, 4 AEs per year of follow-up occurred in the VDZ-treated group and 12 in the anti-TNF-treated group. No severe AEs or discontinuation of treatment were reported. There was no significant difference in terms of the safety between these two groups after adjusting the propensity score (*p* = 0.274) (Table 3). The AEs reported by patients were infectious (pyelonephritis, urinary tract infections, pneumonia or perianal abscesses), dermatological (hair loss, non-specific skin rash or paradoxical psoriasis) and rheumatological (joint pains). Only one (5%) patient experienced a dermatological complication with VDZ, compared with four (8%) in patients treated with anti-TNF (Table 4).

For patients with UC, after adjusting on the propensity score, 31 patients were treated with VDZ and 33 patients with anti-TNF. The occurrence of AEs per year of follow-up was similar between the two groups (*p* = 0.876) (Table 3). The rate of infections in both groups was similar (urinary tract infections, pneumonia or perianal abscesses). In the VDZ group, one patient presented with dermatological complication, whereas, in the anti-TNF group, there were none (Table 5).

## 4. Discussion

Biologic therapy has revolutionized the treatment of moderate-to-severe CD and UC [18,19]. The safety profile in randomized controlled trials and prospective registry studies is generally favorable, with few patients experiencing severe adverse events [18].

Several observational studies have shown that therapeutic management in older patients with IBD is complex, mainly due to a lack of data regarding the safety and efficacy of biologics in older patients [10,13,14,20]. The objective of this study was to assess the safety of VDZ and UST therapy in older patients with IBD.

In our study, there is no significant difference between VDZ and UST therapy in terms of safety for older patients with CD. Asscher et al. assessed the impact of age and comorbidities on safety in VDZ and UST-treated patients with IBD using data from the Dutch ICC Registry. They found no association between age and safety outcomes [21].

For UST, 37% of patients reported adverse events in this study. One of the theoretical advantages of UST is a potentially lower rate of infectious events because of its highly selective action [22]. In our study, this rate was 27%. Garg et al. performed a retrospective study of patients with CD classified as elderly (age ≥ 65 years) at UST initiation [17]. Two patients (5.2%) developed infectious complications, and there was no difference with non-elderly patients.

In our study, the rate of AEs per year was 0.23 in the VDZ group for patients with CD, and 0.37 for patients with UC. There was no difference in terms of the safety between VDZ and UST or anti-TNF. These finding are comparable with published studies, which found no evidence of an increased risk of any adverse event in older patients with IBD treated with VDZ. Yajnik et al. investigated the safety of VDZ in patients stratified by age from the GEMINI trials [14]. Patients ≥ 65 years old reported a generally similar adverse event profile to patients < 65 years old. In a retrospective study that included patients with CD or UC initiating anti-TNF or VDZ therapy ≥ 60 years of age, there were no significant differences in the safety profile between the two therapeutic classes at one year [15].

Because VDZ acts by preventing lymphocytes from reaching target organs through the α4-β7 integrin, as it is involved in the body’s defenses against infection, there is a theoretical risk of an increased frequency of infections in these target organs [23]. Kochar et al. showed that older patients with IBD (≥65 years) treated with VDZ had a lower risk of infection-related hospitalization compared with those initiating anti-TNFs [16]. In our cohort, there was no significant difference in the infection rate between patients treated with VDZ or anti-TNF.

Our study has some limitations. First, related to the retrospective design of the cohort, the selection of the VDZ, UST or anti-TNF agent for treatment was non-random and at the discretion of the treating physician. Second, the assessment of adverse events may have been incomplete due to missing information. As data were from referral medical centers, our findings may not be generalizable to population-based IBD cohorts.

## 5. Conclusions

In conclusion, the therapeutic management of older patients with IBD is challenging, particularly due to the lack of evidence-based guidelines for these patients. This study presents a bicentric retrospective cohort demonstrating that both VDZ and UST therapy are comparably safe in older patients with IBD. With the increase in the number of patients treated after 60 years of age for IBD, there is a need to include older adults with IBD in clinical trials.

## Figures and Tables

**Table 1:** Comparison of baseline characteristics for patients with Crohn’s disease according to treatment.

		Vedolizumab	Ustekinumab	Anti-TNF	
		*n* = 22	*n* = 30	*n* = 63	*p*
Gender *n* (%)	Male	11 (50)	9 (30)	35 (56)	0.068
	Female	11 (50)	21 (70)	28 (44)	
Median age at diagnosis		42	50	60	0.004
Median duration of disease, year		23	15	5	0.0002
Median duration of follow-up		1	1	1	0.447
Perineal disease, *n* (%)		7 (32)	4 (13)	11 (19)	0.247
	Missing			5	
Smoking status, *n* (%)					0.366
	Never Smoked	12 (55)	18 (60)	27 (44)	
	Former Smoker	8 (36)	11 (37)	33 (53)	
	Current smoker	2 (9)	1 (3)	2 (3)	
	Unknown			1	
History of surgery, *n* (%)					0.364
	Yes	5 (50)	14 (74)	10 (56)	
	None				
	Missing	12	11	45	
Previous treatment, *n* (%)					
Anti-TNF					-
	Yes	17 (81)	27 (96)	4 (7)	
	Missing	1	2	3	
Vedolizumab	Yes		8 (27)	0	-
	Missing			3	
Ustekinumab		2 (9)		0	-
	Missing			3	
Corticosteroids	Yes	18 (86)	30 (100)	15 (88)	0.114
	Missing	1		46	
Immunosuppressant *n* (%)	Yes	14 (67)	20 (67)	30 (51)	0.245
	Missing	1		4	
Concomitant treatment, *n* (%)					
Corticosteroids		4 (18)	9	33 (57)	0.002
	Missing			5	
Immunosupressant	Yes	5 (23)	10 (33)	16 (28)	0.701
	Missing			6	
Comorbidities *n* (med)		10 (1,.5)	17 (1)	16 (1.,5)	0.996

med (median), *n* (number), TNF (tumor necrosis factor).

**Table 2:** Comparison of baseline characteristics for patients with ulcerative colitis according to treatment.

		Vedolizumab	Anti-TNF	
		*n* = 31	*n* = 35	*p*
Gender, *n* (%)	Male	21 (68)	20 (57)	0.376
	Female	10 (32)	15 (43)	
Median age at diagnosis		58	57	0.899
Median duration of disease		6	4.5	0.371
Median duration of follow up		1	1	0.040
Smoking status, *n* (%)				
	Never Smoked	17 (55)	8 (23)	0.022
	Former Smoker	12 (39)	25 (71)	
	Current smoker	2 (6)	2 (6)	
History of surgery, *n* (%)				0.219
	Yes	0	1 (9)	
	None	16 (100)	10 (91)	
	Missing	15	24	
Previous treatment, *n* (%)				
Anti-TNF				<0.0001
	Yes	24 (77)	4 (12)	
	Missing	0	2	
Vedolizumab	Yes		0	-
	Missing		2	
Ustekinumab	Yes	0	0	-
	Missing	0	2	
Corticosteroids	Yes	28 (90)	8 (89)	0.900
	Missing	0	26	
Immunosuppressant *n* (%)	Yes	20 (64)	19 (61)	0.793
	Missing	0	4	
Concomitant treatment, *n* (%)				
Corticosteroids	Yes	13 (42)	20 (61)	0.135
	Missing	0	2	
Immunosuppressant	Yes	7 (23)	9 (27)	0.665
	Missing	0	2	
Comorbidities, *n* (med)		16 (2)	8 (2)	0.959

med (median), *n* (number), TNF (tumor necrosis factor).

**Table 3:** Comparative safety in patients treated with vedolizumab, ustekinumab or anti-TNF.

	Vedolizumab	Ustekinumab	Anti-TNF	
	*n* = 21	*n* = 27	*n* = 54	
Number of AE per year of follow up (CD)				p *
Vedolizumab vs. Ustekinumab	0.23	1.03		0.258
Vedolizumab vs. Anti-TNF	0.23		1.02	0.274
Ustekinumab vs. Anti-TNF		1.03	1.02	0.360
	**Vedolizumab**	**Anti-TNF**		
	*n* = 31	*n* = 33		
Number of AE per year of follow up (UC)			p *	
	0.37	0.27	0.876	

* Adjusted comparison test on inverse propensity score weighting. AE (adverse event), CD (Crohn’s disease), TNF (tumor necrosis factor), UC (ulcerative colitis).

**Table 4:** Comparative safety in patients with Crohn’s disease treated with vedolizumab, ustekinumab or anti-TNF therapy.

	Vedolizumab	Ustekinumab	
	*n* = 21	*n* = 27	
Adverse event per year of follow-up, *n* (%)			*p*
Total	4 (19)	10 (37)	0.1737
Dermatological	1	2	
Infectious	3	7	
Allergic	0	1	
Rheumatological	0	0	
Neoplasia	0	0	
Dermatological + Infectious	0	0	
	Vedolizumab	Anti TNF	
	*n* = 21	*n* = 54	
Adverse event per year of follow-up, *n* (%)			*p*
Total	4 (19)	14 (26)	0.5312
Dermatological	1	3	
Infectious	3	4	
Allergic	0	0	
Rheumatological	0	4	
Neoplasia	0	2	
Dermatological + Infectious	0	1	
	Ustekinumab	Anti TNF	
	*n* = 27	*n* = 54	
Adverse event per year of follow-up, *n* (%)			*p*
Total	10 (37)	14 (26)	0.3019
Dermatological	2	3	
Infectious	7	4	
Allergic	1	0	
Rheumatological	0	4	
Neoplasia	0	2	
Dermatological + Infectious	0	1	

**Table 5:** Comparative safety of vedolizumab and anti-TNF in patients with ulcerative colitis.

	Vedolizumab	Anti-TNF	
	*n* = 31 (48%)	*n* = 33 (52%)	
Adverse event per year of follow-up, *n* (%)			*p*
Total	7 (23)	7 (21)	0.895
Dermatological	1	0	
Rheumatological	0	2	
Infectious	3	3	
Other adverse event	2	2	
Dermatological + Infectious	1	0	

## Data Availability

The data underlying this article are available in the article.

## Abbreviations

AEs, adverse events; CD, Crohn’s disease; IBD, inflammatory bowel disease; PYs, patient years; RR, relative risks; TNF, tumor necrosis factor; UC, ulcerative colitis; UST, ustekinumab, VDZ, vedolizumab
